# Local thoracic therapy improve prognosis for stage IV non-small cell lung cancer patients combined with chemotherapy: A Surveillance, Epidemiology, and End Results database analysis

**DOI:** 10.1371/journal.pone.0187350

**Published:** 2017-11-10

**Authors:** Kaitai Liu, Dawei Zheng, GuoDong Xu, Zhennan Du, Shibo Wu

**Affiliations:** 1 Department of Radiation Oncology, Lihuili Hospital, Ningbo Medical Center, Ningbo, China; 2 Department of Thoracic Surgery, Lihuili Hospital, Ningbo Medical Center, Ningbo, China; 3 Department of Respiratory Medicine, Lihuili Hospital, Ningbo Medical Center, Ningbo, China; 4 Department of Respiratory Medicine, The Second Hospital of Yinzhou, Ningbo, China; University of Texas MD Anderson Cancer Center, UNITED STATES

## Abstract

Patients with stage IV non-small cell lung cancer (NSCLC) comprise a heterogeneous group, and the optimal treatment for this group of patients is complex and debatable. We aimed to assess the effect of local thoracic therapy combined with chemotherapy on cancer specific survival (CSS). To evaluate the CSS of four subgroups of patients with stage IV NSCLC according to four different treatment modalities: combined modality of Chemotherapy, Surgery, and Radiation (Chem+Sur+RT), Chemotherapy and Radiation (Chem+RT), Chemotherapy and Surgery (Chem+Sur), and Chemotherapy only (Chem Only) by analyzing the Surveillance, Epidemiology, and End Results (SEER)-registered database. Kaplan-Meier methods were adopted and multivariable Cox regression models were built for the analysis of survival outcomes and risk factors. The 3-year CSS was 33.5% in “Chem+Sur+RT” group, 9.3% in “Chem+RT” group, 42.7% in “Chem+Sur” group and 11.8% in “Chem Only” group, which had significant difference in univariate log-rank test (P<0.001) and multivariate Cox regression (P<0.001). Moreover, we observed significant survival benefits in “Chem+Sur” group in all stage of T/N categories, including stage I, stage II, stage IIIa and stage IIIb (all P<0.001). Multimodality therapy, especially combined thoracic surgery and chemotherapy is associated with dramatically improved prognosis for patients with stage IV NSCLC.

## Introduction

Lung cancer is the leading cause of cancer death in the United States [1]. It is estimated that 116,990 new cases in men and 105,510 cases in women of lung and bronchia caner will be diagnosed, and 84,590 deaths in men and 71,280 in women are estimated to occur because of the disease [2]. Only 18.1% of all patients with lung cancer are alive 5 years or more after diagnosis [3]. Approximately half of all patients with non-small cell lung cancer (NSCLC) present with metastatic disease at the time of diagnosis and this group of patients are considered to be incurable by using the current cancer treatment [4–5]. Moreover, data showed that the predominant pattern of failure in patients with local advanced NSCLC is distant metastatic spread [6–7]. Most patients with stage IV NSCLC receive systemic therapies, including chemotherapy or targeted therapy as the primary treatment in the management of stage IV disease. By rational projection, effective systemic treatment combined with aggressive local therapy may be beneficial for patients with metastatic disease, especially to patients medically fit for the combined modality therapy. Some studies have evaluated the role of local controls in systemic disease with conflicting evidence [8–9]. Actually, patients with stage IV non-small cell lung cancer comprise a heterogeneous group, and the optimal treatment for this group of patients is complex and debatable.

Therefore, we conducted this retrospective study to evaluate the cancer specific survival (CSS) of four groups of patients with stage IV NSCLC by analyzing the Surveillance, Epidemiology, and End Results (SEER)-registered database. Subgroups were conducted according to four different treatment modalities: combined modality of Chemotherapy, Surgery, and Radiation (Chem+Sur+RT), Chemotherapy and Radiation (Chem+RT), Chemotherapy and Surgery (Chem+Sur), and Chemotherapy only (Chem Only).

## Materials and methods

### Patient selection in the SEER database

The SEER, a population-based reporting system, was surveyed for the retrospective collection of data used in the analysis. The SEER program collects and publishes cancer incidence and survival data from 18 population-based cancer registries, covering approximately 28% of the population in the United States. The SEER data contain no identifiers and are publicly available for studies of cancer-based epidemiology and survival analysis.

Cases of lung cancer (C34.0–34.3, C34.8–34.9) diagnosed from 2004 to 2013 were extracted from the SEER database (SEER*Stat 8.3.4) according to the Site Recode classifications. We selected this range because AJCC TMN stage was available since 2004 and patients diagnosed after 2013 were excluded to ensure an adequate follow-up time. Only patients with the stage of “IV” (any T and/or any N, distant metastases M1) and the histological type of non-small cell lung cancer aged from 10 to75 years were included into the current study. Patients were excluded if the chemotherapy record was no/unknown. Other exclusion criteria were as follows: unknown TNM stage, unknown survival months, unknown treatment modality and not the first tumor.

This study was based on the publicly available data from the SEER database and we had got the permission to access these research data (SEER*Stat username: liuk).

### Statistical analysis

Age, sex, race, histological grade, histotype and cancer specific survival (CSS) were extracted from SEER database. CSS was calculated from the date of diagnosis to the date of cancer specific death. Deaths attributed to the lung cancer were treated as events and deaths from other causes were treated as censored observations. The intergroup comparison of clinicopathologic variables were performed with the chi-square test. Survival was analysed using the Kaplan-Meier method [8]. The association between each of the potential prognostic factors and the estimated CSS was tested with the log–rank test [10]. Multivariate analysis was performed using the Cox regression model [11]. The statistical test was two sided and P < 0.05 was considered statistically significant. PASW Statistics 19 (SPSS Inc., Chicago, USA) was used for the statistical analysis.

## Results

### Patient characteristics and treatment pattern features

**Fig 1:**
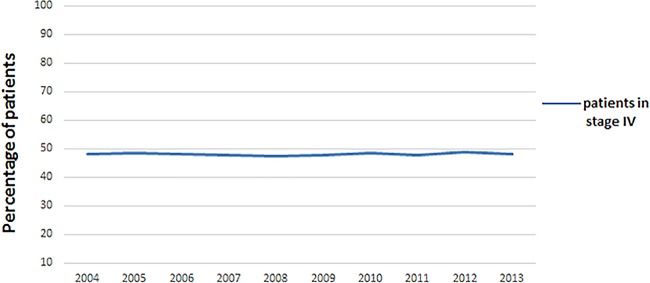
Trend of the proportion of stage IV NSCLC patients from 2004 to 2013.

**Fig 2:**
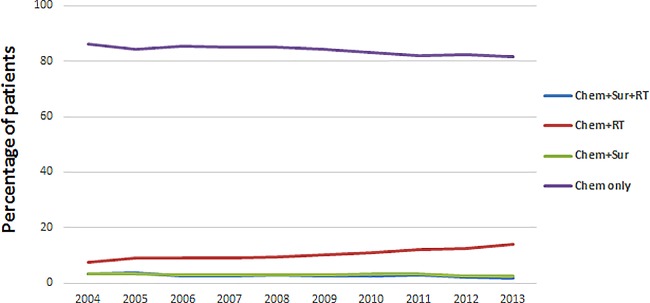
Patterns of care for stage IV NSCLC patients according to treatment modality.

**Fig 3:**
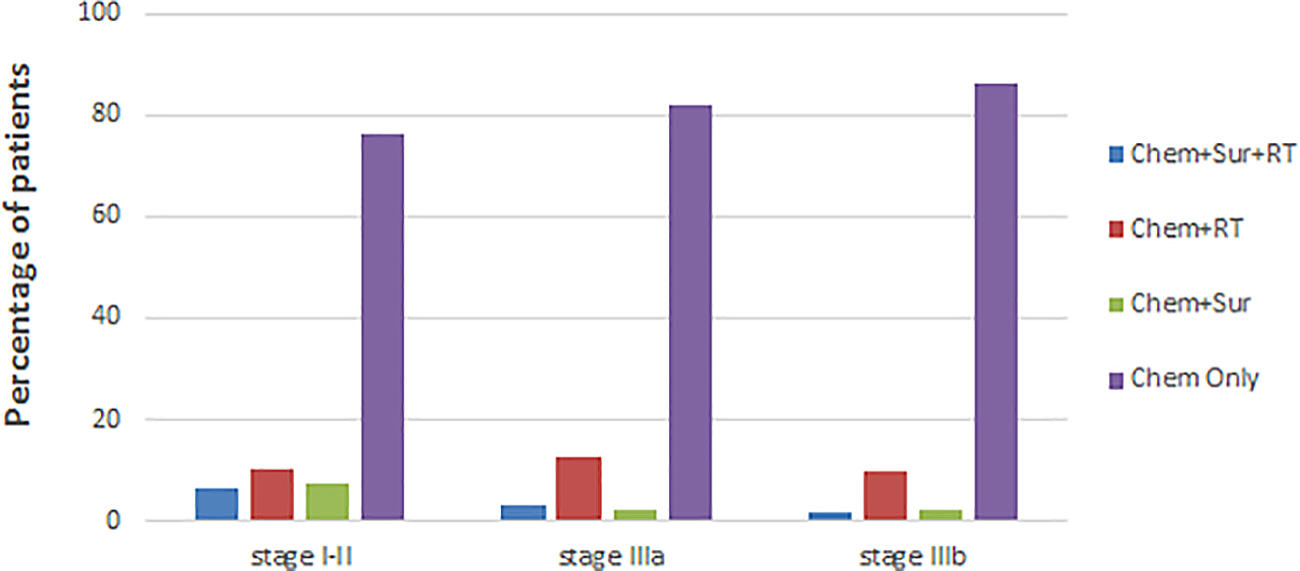
Patterns of care for stage IV NSCLC patients according to stage of T/N.

In all, 45321 patients during the 10-year study period (between 2004 and 2013) in SEER database who met inclusion criteria were identified. Among these patients, 25,114 (55.4%) were males and 20,207 (44.6%) were females. There were 1237 patients in ““Chemotherapy (Chem)+Surgery (Sur)+Radiation (RT)” group, 24,966 patients in “Chem+RT” group, 1339 patients in “Chem+Sur” group, and 17,779 patients in “Chem only” group. The median diagnosis age was 62 years (range, 10–75) and the major race were White (77.7%). Patient demographics and pathological features are summarized in Table 1. Almost half of all patients with NSCLC present with metastatic disease at the time of diagnosis and the trend changed little from 2004 to 2013 (Fig 1). “Chem+RT” was the steadily overwhelming treatment pattern in patients with stage IV non-small cell lung cancer, accounted for more than half of the population. “Chem Only” accounted for almost 40% of care patterns and was the second most common treatment pattern. Obviously, “Chem+Sur+RT” and “Chem+Sur” were the rare option in stage IV NSCLC (Fig 2). Moreover, “Chem+Sur+RT” and “Chem+Sur” were notably more common in these patients with early stage of T/N category. “Chem+RT” and “Chem Only” were most often used in stage IIIa group and stage IIIb group (Fig 3).

**Table 1:** Patient characteristics.

	Total	Chem+Sur+RT	Chem+RT	Chem+Sur	Chem Only	
Variable	n = 45,321	n = 1,237(%)	n = 24,966(%)	n = 1,339(%)	n = 17,779(%)	P value
Sex						<0.001
• Male	25114	644(52.1)	13983(56.0)	661(49.4)	9826(55.3)	
• Female	20207	593(47.9)	10983(44.0)	678(50.6)	7953(44.7)	
Race						<0.001
• White	35194	1028(83.1)	19456(77.9)	1054(78.7)	13656(76.8)	
• Black	6108	131(10.6)	3458(13.9)	160(11.9)	2359(13.3)	
• Other	4019	78(6.3)	2052(8.2)	125(9.4)	1764(9.9)	
Pathological grading						<0.001
• Grade I	1108	45(3.6)	402(1.6)	152(11.4)	509(2.9)	
• Grade II	5056	274(22.2)	2535(10.1)	423(31.6)	1824(10.3)	
• Grade III	13212	605(48.9)	7432(29.8)	506(37.8)	4669(26.3)	
• Grade IV	909	46(3.7)	489(2.0)	38(2.8)	336(1.9)	
• Unknown	25036	267(21.6)	14108(56.5)	220(16.4)	10441(58.6)	
Stage of T/N						<0.001
• Stage I	4374	268(21.7)	2461(9.9)	311(23.2)	1334(7.5)	
• Stage II	2518	179(14.5)	1424(5.7)	185(13.8)	730(4.1)	
• Stage IIIa	10003	317(25.6)	6131(24.6)	227(17.0)	3328(18.7)	
• Stage IIIb	28426	473(38.2)	14950(59.8)	616(46.0)	12387(69.7)	

Abbreviations: Chem: chemotherapy; RT: radiation; Sur: surgery.

### Impact of different treatment patterns on survival outcomes in patients with stage IV non-small cell lung cancer

**Fig 4:**
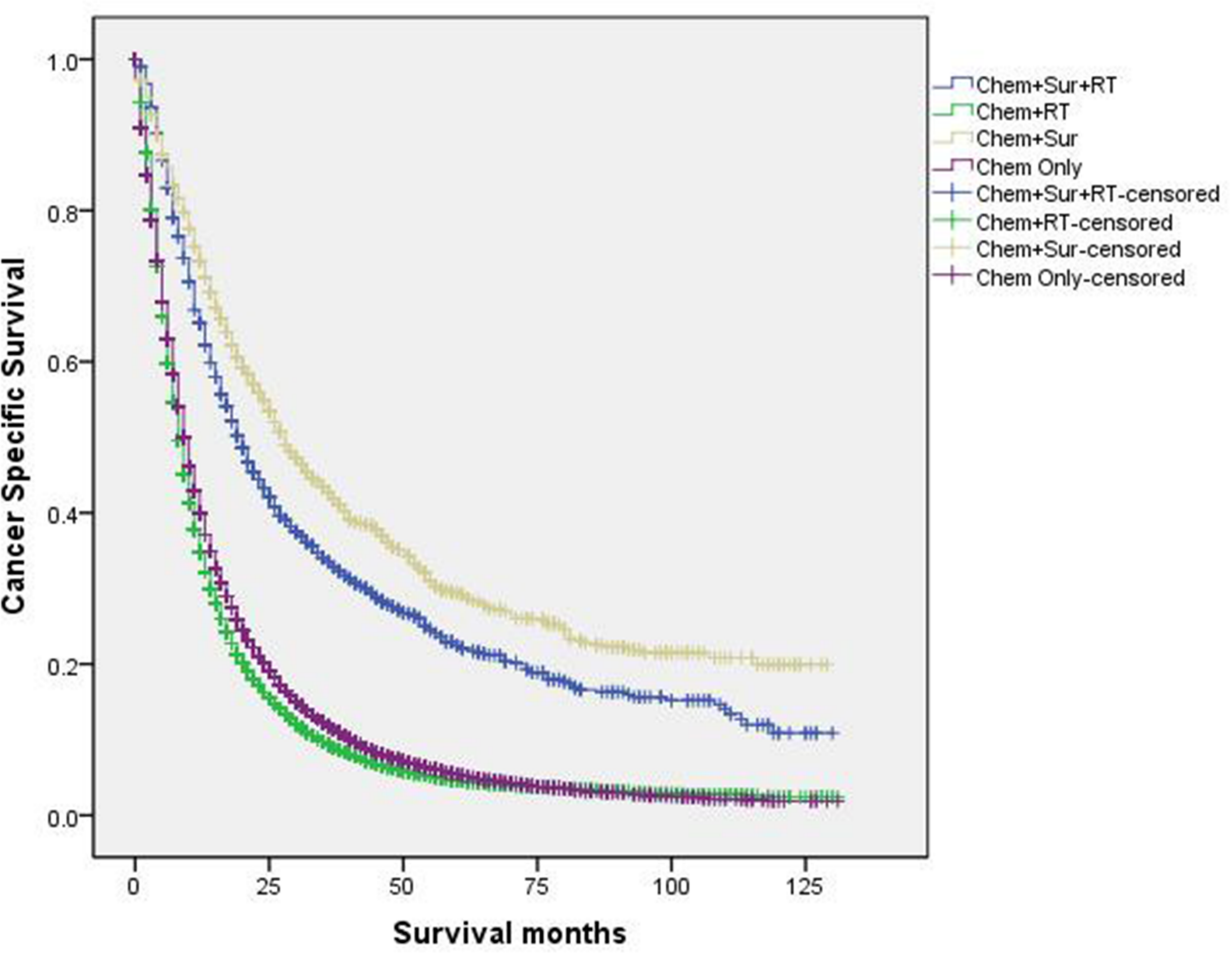
Survival curves in stage IV NSCLC patients according to different treatment modality.

**Fig 5:**
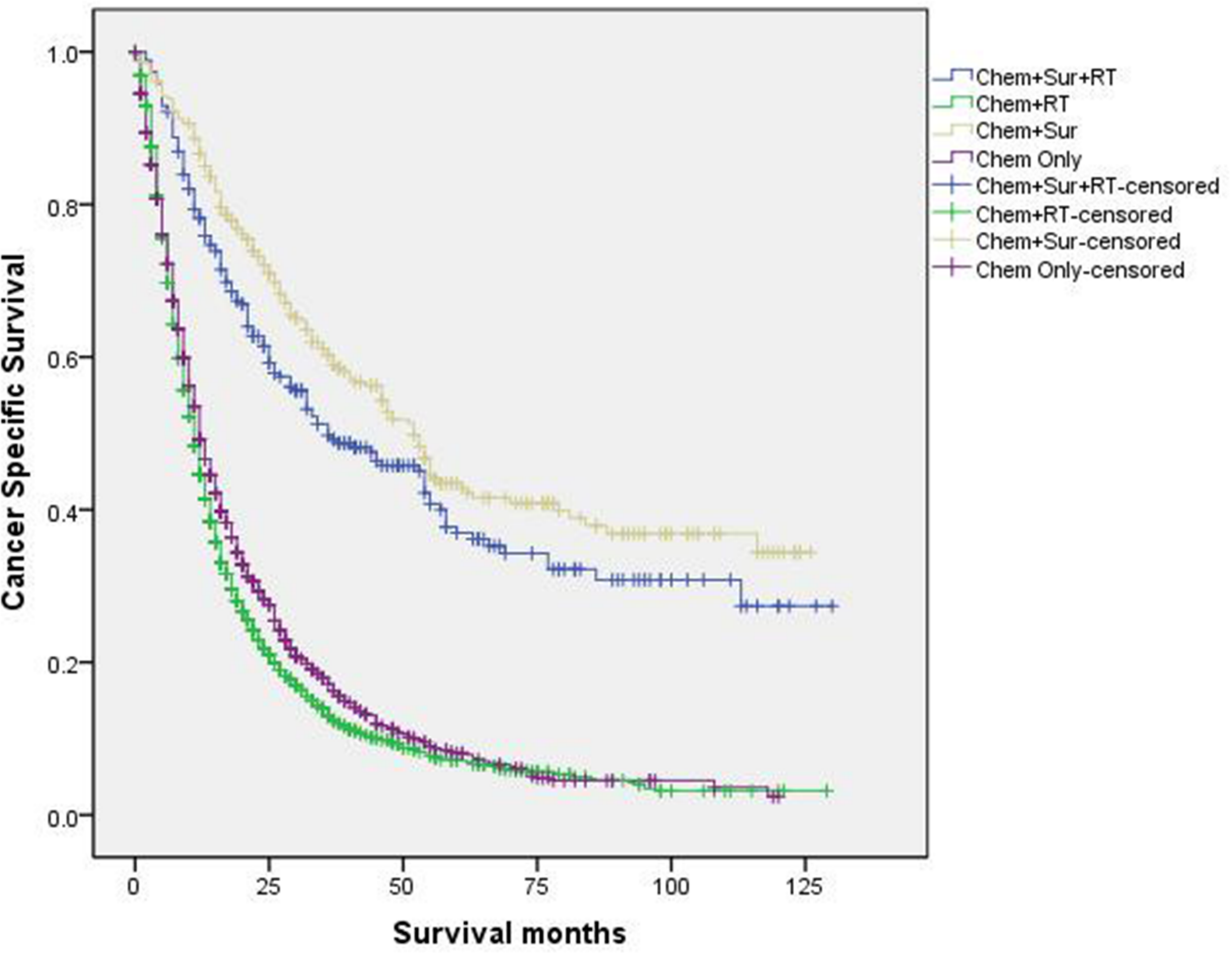
Survival curves in stage IV NSCLC patients according to treatment modality in stage I of T/N category.

**Fig 6:**
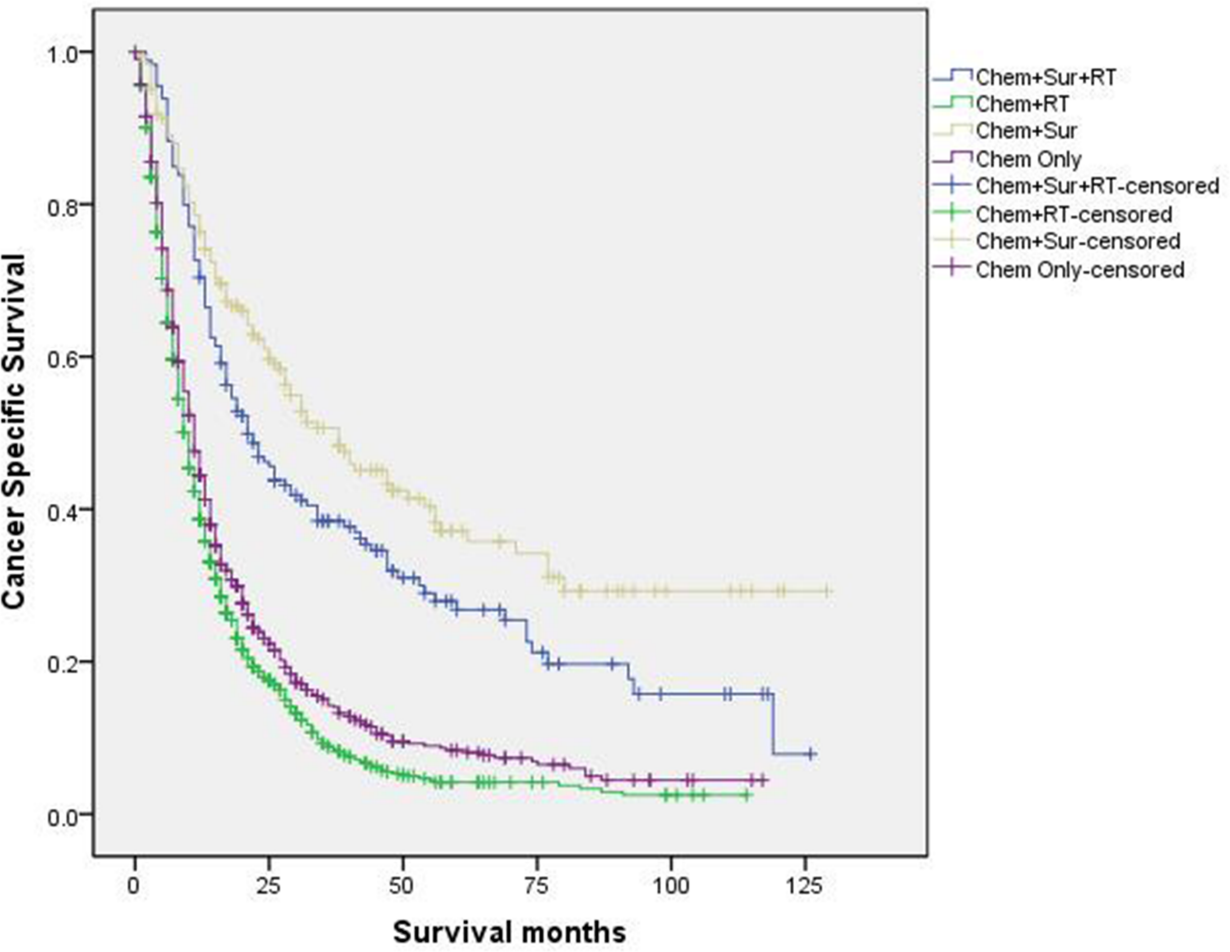
Survival curves in stage IV NSCLC patients according to treatment modality in stage II of T/N category.

**Fig 7:**
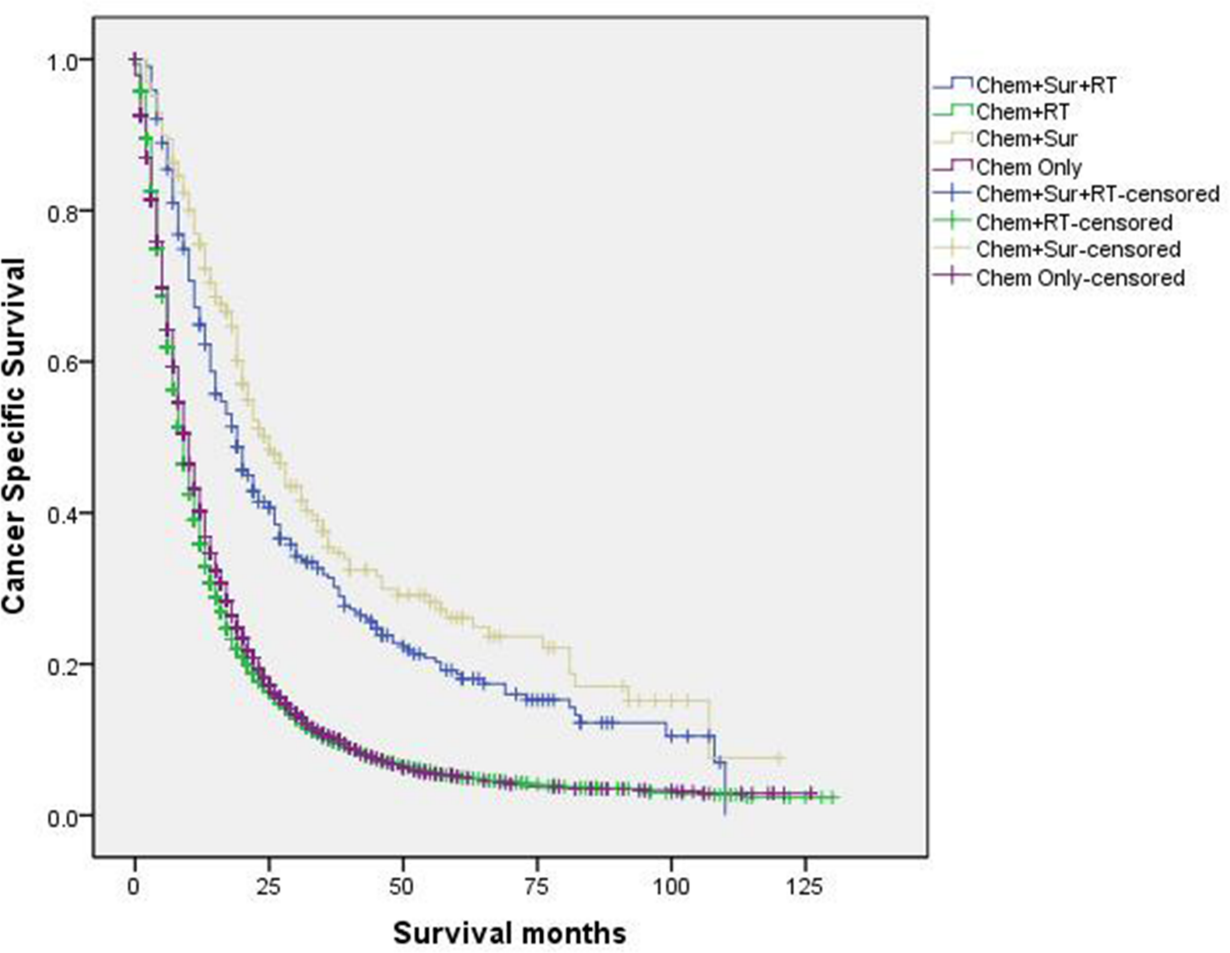
Survival curves in stage IV NSCLC patients according to treatment modality in stage IIIa of T/N category.

**Fig 8:**
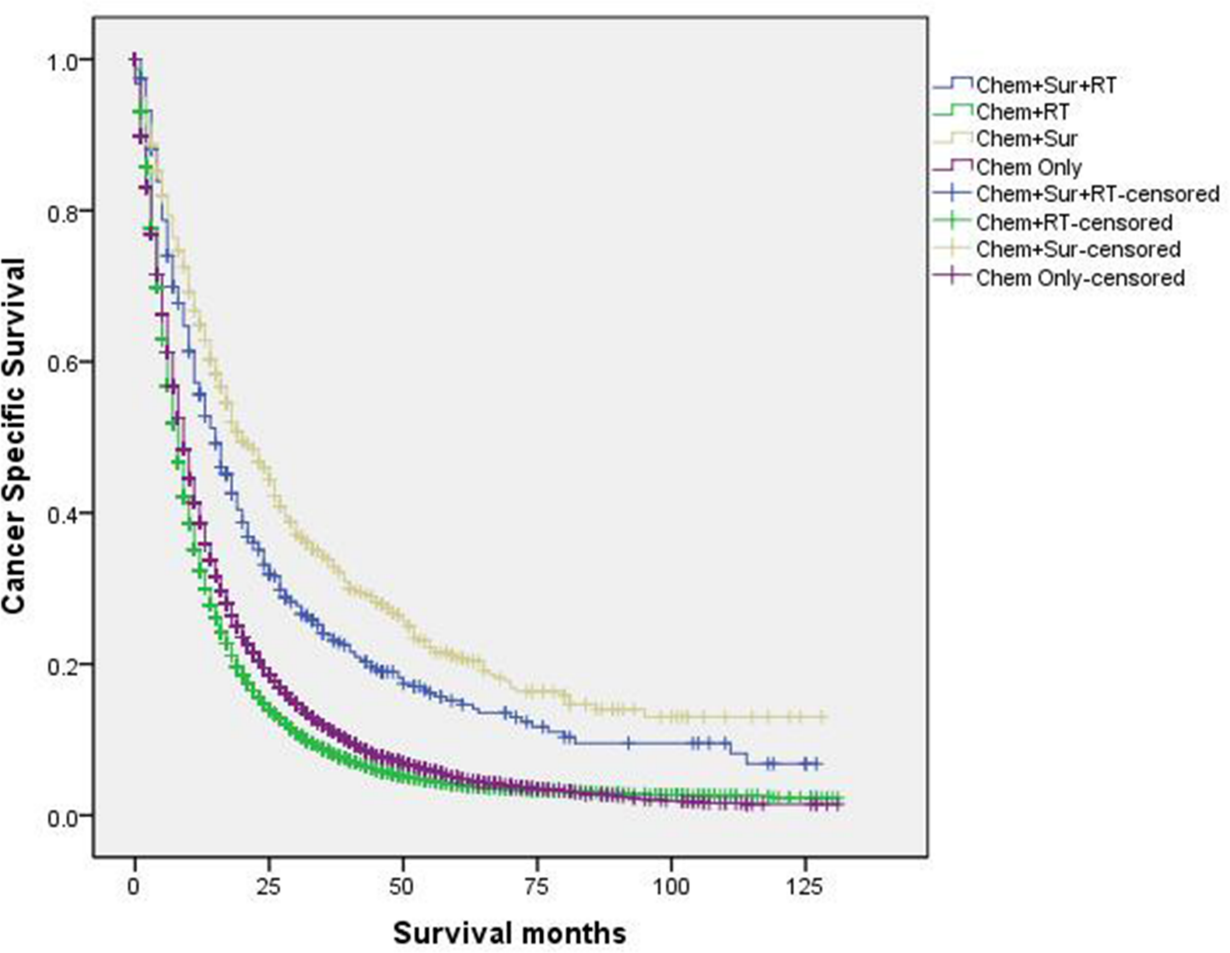
Survival curves in stage IV NSCLC patients according to treatment modality in stage IIIb of T/N category.

When evaluated the 3-year CSS rate of four subgroups in univariate log-rank test, “Chem+Sur” group significantly had the best outcome of 42.7%, “Chem+Sur+RT” group had the second-best one of 33.5%, “Chem+RT” group had the medial 3-year CSS rate of 11.8% and “Chem Only” group had the worst one of 9.3% (P<0.001) (Fig 4). Besides, we conducted survival analyses according to different stage of T/N groups (stage I, stage II, stage IIIa, stage IIIb, Figs 5–8). Results showed that “Chem+Sur” group had statistically better cancer specific survival in all stage of T/N categories (all P<0.001). In univariate analysis, we identified older age, male, white and black race, advanced stage of T/N, higher tumor grade as significantly adverse prognostic factors (all P<0.001) (Table 2). All these factors were confirmed as independent prognostic factors in multivariate analysis by controlling above mentioned factors used Cox regression model (Table 2).

**Table 2:** Univariate and multivariate survival analyses of patients with advanced non-small lung cancer according to various clinicopathological variables.

Variable	n	3-year	Univariate	Multivariate	HR	95% CI
		CSS (%)	P	P		
Age (year)			<0.001	<0.001	1.085	1.064–1.108
• ≤60	19697	12.7				
• >60	25624	11.5				
Sex			<0.001	<0.001	0.812	0.795–0.828
• Male	25114	9.5				
• Female	20207	15.1				
Race			<0.001	<0.001		
• White	35194	11.3		<0.001	1.426	1.375–1.480
• Black	6108	10.7		<0.001	1.377	1.318–1.440
• Other	4019	20.4		ref	ref	ref
Treatment pattern			<0.001	<0.001		
• Chem+Sur+RT	1237	33.5		<0.001	0.542	0.506–0.581
• Chem+RT	24966	9.3		<0.001	1.101	1.078–1.124
• Chem+Sur	1339	42.7		<0.001	0.453	0.422–0.486
• Chem Only	17779	11.8		ref	ref	ref
Stage of T/N			<0.001	<0.001		
• Stage I	4374	20.0		<0.001	0.731	0.706–0.758
• Stage II	2518	15.9		<0.001	0.831	0.794–0.869
• Stage IIIa	10003	11.5		<0.001	0.911	0.889–0.934
• Stage IIIb	28426	10.6		ref	ref	ref
Pathological grading			<0.001	<0.001		
• Grade I	1108	25.2		<0.001	0.724	0.676–0.776
• Grade II	5056	18.0		<0.001	0.854	0.825–0.883
• Grade III	13212	11.1		<0.001	1.072	1.048–1.097
• Grade IV	909	7.7		<0.001	1.255	1.171–1.345
• Unknown	25036	10.8		ref	ref	ref

Abbreviations: CSS: cancer specific survival; Chem: chemotherapy; RT: radiation; Sur: surgery; ref: reference; HR: hazard ratio; CI: confidence interval.

## Discussion

Non-small cell lung cancer is the leading cause of cancer-related death worldwide [12]. Patients with stage IV NSCLC extremely have a poor prognosis, with a median survival of 8–10 months, even though the traditional platinum-based doublet chemotherapy improve quality of life and extend survival [13]. In the present study, we found patients with stage IV made up about 50% of the population of NSCLC and the proportion fell little from 2004 to 2013. For the past few years, the investigation of molecular signal pathways and the advances of systemic therapies targeted at some mutation sites such as sensitizing EGFR mutations, ALK fusion oncogene and ROS1 and RET gene rearrangements have transformed the management of metastatic NSCLC, particularly in specific few subgroups of patients, resulting in significantly longer survival [14–19]. Even so, how to improve the survival remains the major challenge in the management of patients with metastatic NSCLC. According to the National Comprehensive Cancer Network (NCCN) guidelines, systemic chemotherapy and targeted therapy are the recommended first-line treatment in patients with stage IV NSCLC [20]. However, stage IV NSCLC represents a heterogeneous stage grouping, with regard to the extent of thoracic disease, extent of disease spread, performance status and other prognostic factors, the optimal treatment for this group of patients is complex and debatable.

Kyle e. rusthoven et al. evaluated the pattern of failure (POF) after first-line systemic therapy in advanced non-small cell lung cancer [21]. They found that among all eligible patients, 64% presented first extra-cranial progression as local for lesions known prior to treatment only. Distant for new lesions only was found in 9% and 27% patients presented both local and distant progression. They concluded that the predominant POF in patients with advanced NSCLC after first-line systemic therapy was local-only. Thus in theory, combined appropriate local thoracic therapy with systemic therapy may be beneficial for patients with metastatic disease, especially to patients with good performance status and localized extent of thoracic disease.

Chermiti Ben Abdallah F and colleagues observed better survival in patients aged less than 60 years, having better performance status and patients who received specific anti-tumor treatment with surgery and combined chemotherapy and radiation [22]. Jihen Ben Amar et al. assessed overall survival and prognostic factors in patients with locally advanced or metastatic NSCLC [23]. They retrospectively studied 180 patients with non-small cell lung cancer between January 2007 and December 2014. The mean age was 61.5 years with a male predominance (93.3%). There were 55.6% patients present stage IV disease. The median overall survival was 6 months. But patients who underwent surgery and combined chemotherapy and radiation were observed in significantly prolonged overall survival of 61 and 10 months, respectively. Some studies showed that surgical treatment of primary tumor could prove beneficial in non-small cell lung cancer patients with synchronous brain metastases and oligometastatic diseases. Peter S. Billing found patients with brain metastases underwent resection of primary lung disease had good overall survival at 1, 2, and 5 years was 64.3%, 54.0% and 21.4%, respectively [24]. Yoshinaga Y evaluated the effectiveness of surgical treatment for non-small cell lung cancer patients with synchronous brain metastases (stage IV) [25]. Similarly, they found median survival time was 331 days in patients received both lung and brain resection, 151 days in patients received lung resection plus gamma knife therapy and 92 days in patients received brain resection. Jinyuan He et al. investigated the effect of local surgery for NSCLC with pulmonary oligometastasis [26]. The median survival time of local surgery group and systematic chemotherapy group were 37 and 11.6 months respectively, 5-year survival rates were 18.2 and 9.1% respectively (p < 0.05). Results showed that local surgery significantly prolonged the overall survival time and 5-year survival rate of primary NSCLC with pulmonary oligometastasis. Nuria M. Novoa et al. reviewed the surgical management of oligometastatic non-small cell lung cancer [27]. They concluded that with accurate staging of the tumor the role for an aggressive local treatment would benefit a large group of patients with stage IV NSCLC. Meanwhile, strict selection of patients according to T and N status, completeness of resection of the primary tumor, adenocarcinoma histology and location of the metastatic lesion should be considered to delimit a group of favorable response to a curative-intent surgical treatment.

Research has showed that radiotherapy was effective for improvement of symptoms resulting from intrathoracic disease as a palliative treatment and in approximately one third of patients, improves global quality of life [28]. Still, even more controversial was whether palliative RT has impact on survival or not, but results were conflicting, because some studies found that higher-dose radiation leads to prolonged survival, some concluded equivalence and one showed that RT worsened survival [29]. Tommy Sheu retrospectively analyzed comprehensive local therapy (CLT) influencing survival in 90 patients with NSCLC presenting within 3 synchronous metastatic lesions between 1998 and 2012 [30]. Local therapies to primary tumor were as follows: 9% in surgery, 3% in SARB and 68% in fractionated RT. They observed statistically significant benefits in overall survival (27.1 vs 13.1 months) and progression free survival (11.3 months vs 8.0 months) in CLT group. Author did not find a clear survival benefit for patients with metastatic disease limited to the brain, suggesting that if other clinical indicators favor CLT, the presence of multi-organ or non-brain/non-adrenal involvement should not be considered an intrinsic contraindication for pursuing aggressive therapy. Moreover, good performance status was associated with improved survival. This association has been demonstrated in several studies of aggressive local therapy for oligometastatic disease. The simplest explanation for this observation is that patients with good performance status can tolerate more aggressive therapy. They concluded that CLT should be considered for oligometastatic NSCLC patients with favorable performance status regardless of the distribution of disease sites. A multicenter, randomised, controlled, phase 2 study assessed the effect of local consolidative therapy on progression-free survival in patients with oligometastatic non-small-cell lung cancer [31]. Results showed the median progression-free survival in the local consolidative therapy group was 11.9 months versus 3.9 months in the maintenance treatment group. Adverse events were similar between groups, with no grade 4 adverse events or deaths due to treatment. Authors suggested that aggressive local therapy should be further explored in phase 3 trials. A multicenter phase 2 study from PPRA-RTOG China investigated the role of thoracic three-dimensional radiation therapy (3D-RT) with concurrent chemotherapy for newly diagnosed stage IV non-small cell lung cancer. The study concluded that thoracic 3D-RT to the primary tumor with concurrent chemotherapy for stage IV NSCLC was effective and tolerable, especially for local control. [32].

In agreement with these findings, our study demonstrated combined local thoracic therapy especially surgery and chemotherapy could significantly improve prognosis for stage IV NSCLC in all T/N categories, including stage I group, stage II group, stage IIIa group and stage IIIb group. Univariate and multivariate analyses showed T/N stage was an independent prognostic factor. Furthermore, our study showed that combined surgery and radiation as thoracic treatment only improved relatively little survival. In our opinion, for stage IV NSCLC patients the triple therapy maybe excessive because of the intrinsic poor prognosis and ordinary performance status. Besides, we found combined radiation as thoracic therapy did not increase survival. To our minds, the thoracic radiation may be useful specifically for oligometastatic non-small-cell lung cancer but we can not pick out this population because of the limitation of SEER database. Another important reason may be that patients in our study might undergo old technology on a much more regular basis because of the study period, even though the SEER database lack of data in radiation parameters. We can reasonably infer that local thoracic radiation may play more important role in stage IV NSCLC with advanced technology, such as intensity modulated radiotherapy (IMRT), image-guided radiation therapy (IGRT) and stereotactic body radiotherapy (SBRT).

Although this is a large population-based study, it has several potential limitations. First, the SEER registry does not collect information on the comorbidities, nutritional status, or performance status of the patients. One reason that the advanced patients probably underwent less aggressive treatment may be due to comorbidities and poor performance status. Thus, the final results of our study might be confounded because these confounding factors were not able to be adjusted. Second, our study is the lack of data in the SEER registry on the regimen of chemotherapy, the sequence of local therapy and chemotherapy, dose and technology of radiation, resulting in a potentially significant confounder in the current study. Finally, the current analysis of the nonrandomized patient population could not exclude the possibility of selection bias. However, our study has its convincing power for its larger population based study.

In conclusion, multimodality therapy, especially combined thoracic surgery and chemotherapy is associated with dramatically improved prognosis for patients with stage IV NSCLC, especially to patients medically fit for the combined modality therapy. Ideally, randomized studies should be performed.
