# Correction: Local thoracic therapy improve prognosis for stage IV non-small cell lung cancer patients combined with chemotherapy: A Surveillance, Epidemiology, and End Results database analysis

**DOI:** 10.1371/journal.pone.0196618

**Published:** 2018-04-24

**Authors:** Kaitai Liu, Dawei Zheng, GuoDong Xu, Zhennan Du, Shibo Wu

There are errors in the author affiliations. The affiliations should appear as shown here: Kaitai Liu^1^, Dawei Zheng^2^, GuoDong Xu^2^, Zhennan Du^3^, Shibo Wu^4^.

**1** Department of Radiation Oncology, Lihuili Hospital, Ningbo Medical Center, Ningbo, China, **2** Department of Thoracic Surgery, Lihuili Hospital, Ningbo Medical Center, Ningbo, China, **3** Department of Respiratory Medicine, The Second Hospital of Yinzhou, Ningbo, China, **4** Department of Respiratory Medicine, Lihuili Hospital, Ningbo Medical Center, Ningbo, China.
